# Supplementary material for: Translation attenuation by minocycline enhances longevity and proteostasis in old post-stress-responsive organisms
Source: eLife. 2018 Nov 27;7:e40314. doi: 10.7554/eLife.40314 (PMC6257811; doi:10.7554/eLife.40314)
Supplement: Figure 1—source data 1. [file elife-40314-fig1-data1.docx]

**Figure 1—source data 1. Summary of N2 lifespan data, related to Figure 1.**

| **Exp. 1** |  | **Minocycline Concentration [μM]** | | | | | |
| --- | --- | --- | --- | --- | --- | --- | --- |
| **Strain** |  | **0** | **2** | **10** | **50** | **100** | **200** |
| N2 / day 1 | Change in lifespan [%] | - | -6 | -2 | 5 | 36 | 43 |
|  | P-value | - | .17 | .57 | .06 | 2.6E-7 | 8.7E-18 |
|  | Mean Lifespan [days] | 27.5 | 25.7 | 27.0 | 29.0 | 37.3 | 39.4 |
|  | Number of animals | 61 | 52 | 74 | 76 | 68 | 48 |
| N2 / day 8 | Change in lifespan [%] | - | 3 | 2 | 10 | 17 | 24 |
|  | P-value | - | .55 | .79 | .01 | 1.6E-4 | 1.8E-8 |
|  | Mean Lifespan [days] | 25.6 | 26.3 | 26.1 | 28.1 | 30.0 | 31.8 |
|  | Number of animals | 81 | 65 | 71 | 72 | 61 | 70 |
| **Exp. 2** |  | **Minocycline Concentration [μM]** | | | | | |
| **Strain** |  | **0** | **2** | **10** | **50** | **100** | **200** |
| N2 / day 1 | Change in lifespan [%] | - | 0 | -1 | 6 | 33 | 12 |
|  | P-value | - | .88 | .87 | .05 | 4.7E-9 | 2.2E-4 |
|  | Mean Lifespan [days] | 23.8 | 23.7 | 23.5 | 25.2 | 31.7 | 26.6 |
|  | Number of animals | 71 | 63 | 100 | 70 | 55 | 71 |
| N2 / day 8 | Change in lifespan [%] | - | 5 | 6 | 5 | 16 | 19 |
|  | P-value | - | .40 | .19 | .07 | 1.4E-4 | 2.5E-8 |
|  | Mean Lifespan [days] | 22.3 | 23.4 | 23.7 | 23.5 | 25.8 | 26.6 |
|  | Number of animals | 81 | 79 | 58 | 64 | 76 | 63 |
| **Exp. 3** |  | **Minocycline Concentration [μM]** | | | | | |
| **Strain** |  | **0** | **2** | **10** | **50** | **100** | **200** |
| N2 / day 1 | Change in lifespan [%] | - | -2 | 7 | 43 | 51 | 10 |
|  | P-value | - | .87 | .01 | 6.1E-14 | 4.1E-13 | 4.3E-3 |
|  | Mean Lifespan [days] | 21.6 | 21.3 | 23.1 | 31.0 | 32.4 | 23.8 |
|  | Number of animals | 54 | 71 | 72 | 42 | 67 | 52 |
| N2 / day 8 | Change in lifespan [%] | - | -15 | 3 | 6 | 10 | 0 |
|  | P-value | - | 3.5E-4 | .27 | .03 | 1.1E-3, | .48 |
|  | Mean Lifespan [days] | 21.3 | 18.1 | 22.0 | 22.6 | 23.5 | 21.3 |
|  | Number of animals | 51 | 21 | 57 | 58 | 67 | 66 |
| **Exp. 4** |  | **Minocycline Concentration [μM]** | | | | | |
| **Strain** |  | **0** | **2** | **10** | **50** | **100** | **200** |
| N2 / day 1 | Change in lifespan [%] | - | -5 | -3 | 6 | 30 | 14 |
|  | P-value | - | .29 | .21 | 4.8E-3 | 6.0E-15 | 1.1E-5 |
|  | Mean Lifespan [days] | 27 | 25.8 | 26.2 | 28.6 | 35.0 | 30.8 |
|  | Number of animals | 71 | 69 | 72 | 71 | 73 | 67 |
| N2 / day 8 | Change in lifespan [%] | - | -6 | -3 | -3 | 6 | 10 |
|  | P-value | - | .03 | .52 | .36 | .07 | 3.0E-3 |
|  | Mean Lifespan [days] | 26.9 | 25.3 | 26.1 | 26.2 | 28.6 | 29.8 |
|  | Number of animals | 70 | 68 | 71 | 71 | 72 | 70 |
| **Exp. 5** |  | **Minocycline Concentration [μM]** | | | | | |
| **Strain** |  | **0** | **2** | **10** | **50** | **100** | **200** |
| N2 / day 1 | Change in lifespan [%] | - | 1 | 1 | 33 | 48 | 30 |
|  | P-value | - | .71 | .67 | 1.8E-16 | 1.6E-21 | 7.4E13 |
|  | Mean Lifespan [days] | 21.7 | 21.9 | 22.0 | 29.0 | 32.2 | 28.2 |
|  | Number of animals | 64 | 80 | 63 | 81 | 81 | 64 |
| N2 / day 8 | Change in lifespan [%] | - | 3 | 7 | 13 | 22 | 17 |
|  | P-value | - | .37 | .04 | 5.9E-4 | 5.5E-11 | 3.3E-6 |
|  | Mean Lifespan [days] | 21.1 | 21.9 | 22.7 | 23.8 | 25.7 | 24.8 |
|  | Number of animals | 79 | 80 | 73 | 81 | 56 | 81 |

All experiments conducted with dead, γ-irradiated dead bacteria (OP50)
