# Supplementary material for: Translation attenuation by minocycline enhances longevity and proteostasis in old post-stress-responsive organisms
Source: eLife. 2018 Nov 27;7:e40314. doi: 10.7554/eLife.40314 (PMC6257811; doi:10.7554/eLife.40314)
Supplement: Figure 2—source data 1. [file elife-40314-fig2-data1.docx]

**Figure 2—source data 1. Summary of lifespan data for strains carrying mutations in regulators of stress, proteostasis, autophagy and lysosomal responses, related to Figure 2.**

| **Exp. 1** |  | **Minocycline Concentration [μM]** | | |
| --- | --- | --- | --- | --- |
| **Strain** |  | **0** | **100** | **200** |
| *xbp-1 (xc12)* | Change in lifespan [%] | - | 17 | - |
|  | P-value | - | .02 | - |
|  | Mean Lifespan [days] | 18.2 | 21.2 | - |
|  | Number of animals | 40 | 56 | - |
| *skn-1 (zj15)* | Change in lifespan [%] | - | 34 | 25 |
|  | P-value | - | 1.4E-6 | 6.1E-5 |
|  | Mean Lifespan [days] | 20.1 | 26.9 | 25.2 |
|  | Number of animals | 43 | 39 | 39 |
| *hsf-1 (sy441)* | Change in lifespan [%] | - | 159 | - |
|  | P-value | - | 9.2E-13 | - |
|  | Mean Lifespan [days] | 12.0 | 31.1 | - |
|  | Number of animals | 70 | 72 | - |
| *atfs-1 (tm4525)* | Change in lifespan [%] | - | 56 | - |
|  | P-value | - | 1.0E-10 | - |
|  | Mean Lifespan [days] | 22.9 | 35.7 | - |
|  | Number of animals | 59 | 69 | - |
| *daf-16 (mu86)* | Change in lifespan [%] | - | 47 | - |
|  | P-value | - | 2.3E-8 | - |
|  | Mean Lifespan [days] | 15.1 | 22.3 | - |
|  | Number of animals | 46 | 39 | - |
| *unc-51 (e369)* | Change in lifespan [%] | - | 52 | - |
|  | P-value | - | 1.6E-12 | - |
|  | Mean Lifespan [days] | 14.3 | 21.7 | - |
|  | Number of animals | 138 | 82 | - |
| *hlh-30 (tm1978)* | Change in lifespan [%] | - | 32 | - |
|  | P-value | - | 6.7E-5 | - |
|  | Mean Lifespan [days] | 10.7 | 14.2 | - |
|  | Number of animals | 80 | 56 | - |
| **Exp. 2** |  | **Minocycline Concentration [μM]** | | |
| **Strain** |  | **0** | **100** | **200** |
| *xbp-1 (xc12)* | Change in lifespan [%] | - | 21 | - |
|  | P-value | - | .04 | - |
|  | Mean Lifespan [days] | 19.9 | 24.0 | - |
|  | Number of animals | 60 | 65 | - |
| *skn-1 (zj15)* | Change in lifespan [%] | - | 12 | 29 |
|  | P-value | - | 3.7E-4 | 3.1E-4 |
|  | Mean Lifespan [days] | 21.3 | 23.9 | 27.5 |
|  | Number of animals | 35 | 37 | 41 |
| *hsf-1 (sy441)* | Change in lifespan [%] | - | 81 | - |
|  | P-value | - | 1.6E-24 | - |
|  | Mean Lifespan [days] | 12.8 | 23.1 | - |
|  | Number of animals | 68 | 63 | - |
| *atfs-1 (tm4525)* | Change in lifespan [%] | - | 66 | - |
|  | P-value | - | 5.5E-8 | - |
|  | Mean Lifespan [days] | 16.7 | 27.7 | - |
|  | Number of animals | 72 | 77 | - |
| *daf-16 (mu86)* | Change in lifespan [%] | - | 20 | - |
|  | P-value | - | 1.5E-4 | - |
|  | Mean Lifespan [days] | 14.0 | 16.9 | - |
|  | Number of animals | 43 | 49 | - |
| *unc-51 (e369)* | Change in lifespan [%] | - | 24 | - |
|  | P-value | - | 2.6E-10 | - |
|  | Mean Lifespan [days] | 16.3 | 20.3 | - |
|  | Number of animals | 64 | 65 | - |
| *hlh-30 (tm1978)* | Change in lifespan [%] | - | 17 | - |
|  | P-value | - | 1.0E-3 | - |
|  | Mean Lifespan [days] | 16.2 | 18.5 | - |
|  | Number of animals | 80 | 79 | - |
| **Exp. 3** |  | **Minocycline Concentration [μM]** | | |
| **Strain** |  | **0** | **100** | **200** |
| *xbp-1 (xc12)* | Change in lifespan [%] | - | 15 | - |
|  | P-value | - | 2.36E-6 | - |
|  | Mean Lifespan [days] | 15.6 | 17.9 | - |
|  | Number of animals | 218 | 225 | - |
| *skn-1 (zj15)* | Change in lifespan [%] | - | 19 | 74 |
|  | P-value | - | .05 | 1.2E-14 |
|  | Mean Lifespan [days] | 15.7 | 18.7 | 27.6 |
|  | Number of animals | 155 | 61 | 41 |
| *hsf-1 (sy441)* | Change in lifespan [%] | - | 100 | - |
|  | P-value | - | 1.7E-24 | - |
|  | Mean Lifespan [days] | 16.2 | 32.4 | - |
|  | Number of animals | 67 | 58 | - |
| *atfs-1 (tm4525)* | Change in lifespan [%] | - | 13 | - |
|  | P-value | - | .04 | - |
|  | Mean Lifespan [days] | 31.4 | 35.3 | - |
|  | Number of animals | 57 | 70 | - |
| *daf-16 (mu86)* | Change in lifespan [%] | - | 34 | - |
|  | P-value | - | 1.8E-19 | - |
|  | Mean Lifespan [days] | 17.6 | 23.5 | - |
|  | Number of animals | 97 | 62 | - |
| *unc-51 (e369)* | Change in lifespan [%] | - | 65 | - |
|  | P-value | - | 2.2E-14 | - |
|  | Mean Lifespan [days] | 15.6 | 25.8 | - |
|  | Number of animals | 107 | 75 | - |
| *hlh-30 (tm1978)* | Change in lifespan [%] | - | 13 | - |
|  | P-value | - | .15 | - |
|  | Mean Lifespan [days] | 12.6 | 14.3 | - |
|  | Number of animals | 69 | 73 | - |

All experiments conducted with γ-irradiated dead bacteria (OP50)
