# Supplementary material for: Translation attenuation by minocycline enhances longevity and proteostasis in old post-stress-responsive organisms
Source: eLife. 2018 Nov 27;7:e40314. doi: 10.7554/eLife.40314 (PMC6257811; doi:10.7554/eLife.40314)
Supplement: Figure 5—source data 1. [file elife-40314-fig5-data1.docx]

**Figure 5—source data 1. Summary of *rsks-1* and *ncl-1* lifespan data, related to Figure 5 and Figure 5—figure supplement 1.**

| **Exp. 1** |  | **Minocycline Concentration [μM]** | | | | | |
| --- | --- | --- | --- | --- | --- | --- | --- |
| **Strain** |  | **0** | **2** | **10** | **50** | **100** | **200** |
| *rsks-1 (ok1255)* | Change in lifespan [%] | - | -10 | 3 | 38 | 98 | 61 |
|  | P-value | - | .02 | .93 | 2.6E-7 | 3.8E-30 | 8.2E-11 |
|  | Mean Lifespan [days] | 20.9 | 18.8 | 21.6 | 28.8 | 41.3 | 33.5 |
|  | Number of animals | 75 | 57 | 77 | 72 | 81 | 47 |
| *ncl-1 (e1865)* | Change in lifespan [%] | - | -4 | 5 | 18 | 26 | 12 |
|  | P-value | - | .41 | .38 | 7.7E-6 | 4.0E-11 | .01 |
|  | Mean Lifespan [days] | 22.5 | 21.5 | 23.6 | 26.5 | 28.4 | 25.2 |
|  | Number of animals | 87 | 68 | 78 | 83 | 76 | 63 |
| N2 | Change in lifespan [%] | - | -6 | -2 | 5 | 36 | 43 |
|  | P-value | - | .17 | .57 | .06 | 2.6E-7 | 8.7E-18 |
|  | Mean Lifespan [days] | 27.5 | 25.7 | 27.0 | 29.0 | 37.3 | 39.4 |
|  | Number of animals | 61 | 52 | 74 | 76 | 68 | 48 |
| **Exp. 2** |  | **Minocycline Concentration [μM]** | | | | | |
| **Strain** |  | **0** | **2** | **10** | **50** | **100** | **200** |
| *rsks-1 (ok1255)* | Change in lifespan [%] | - | -12 | 9 | 32 | 55 | 80 |
|  | P-value | - | .02 | 1.2E-3 | 2.9E-13 | 3.0E-22 | 9.1E-20 |
|  | Mean Lifespan [days] | 20.2 | 17.8 | 22.1 | 26.8 | 31.3 | 36.4 |
|  | Number of animals | 46 | 61 | 65 | 56 | 65 | 60 |
| *ncl-1 (e1865)* | Change in lifespan [%] | - | -3 | 5 | 18 | 20 | 15 |
|  | P-value | - | .49 | .11 | 1.6E-7 | 5.6E-7 | 2.9E-6 |
|  | Mean Lifespan [days] | 19.2 | 18.6 | 20.1 | 22.6 | 23.0 | 22.1 |
|  | Number of animals | 71 | 66 | 53 | 99 | 75 | 81 |
| N2 | Change in lifespan [%] | - | -2 | 7 | 43 | 29 | 10 |
|  | P-value | - | .87 | 8.4E-3 | 6.1E-14 | 1.3E-8 | 4.3E-3 |
|  | Mean Lifespan [days] | 21.6 | 21.3 | 23.1 | 31.0 | 27.9 | 23.8 |
|  | Number of animals | 54 | 71 | 72 | 42 | 62 | 52 |
| **Exp. 3** |  | **Minocycline Concentration [μM]** | | | | | |
| **Strain** |  | **0** | **2** | **10** | **50** | **100** | **200** |
| *rsks-1 (ok1255)* | Change in lifespan [%] | - | -4 | 14 | 24 | 56 | 36 |
|  | P-value | - | .14 | .06 | 6.0E-5 | 8.5E-21 | 9.2E-11 |
|  | Mean Lifespan [days] | 23.7 | 22.8 | 27.2 | 29.4 | 37.0 | 32.3 |
|  | Number of animals | 51 | 71 | 70 | 63 | 81 | 75 |
| *ncl-1 (e1865)* | Change in lifespan [%] | - | -1 | 5 | 10 | 11 | -2 |
|  | P-value | - | .31 | .23 | .01 | .01 | .35 |
|  | Mean Lifespan [days] | 18.8 | 18.6 | 19.8 | 20.6 | 20.8 | 18.4 |
|  | Number of animals | 59 | 73 | 65 | 70 | 84 | 68 |
| N2 | Change in lifespan [%] | - | -3 | 2 | 14 | 22 | 16 |
|  | P-value | - | .47 | .57 | .01 | 1.2E-5 | 1.3E-4 |
|  | Mean Lifespan [days] | 24.6 | 23.8 | 25.2 | 27.9 | 30.0 | 28.6 |
|  | Number of animals | 77 | 64 | 64 | 53 | 46 | 71 |
| **Exp. 4** |  | **Minocycline Concentration [μM]** | | | | | |
| **Strain** |  | **0** | **2** | **10** | **50** | **100** | **200** |
| *rsks-1 (ok1255)* | Change in lifespan [%] | - | -6 | 0 | 22 | 34 | 16 |
|  | P-value | - | .11 | .59 | 3.3E-7 | 3.4E-15 | 3.4E-6 |
|  | Mean Lifespan [days] | 25.8 | 24.2 | 25.7 | 31.5 | 34.5 | 30.0 |
|  | Number of animals | 56 | 66 | 45 | 72 | 75 | 79 |
| *ncl-1 (e1865)* | Change in lifespan [%] | - | -7 | -4 | 5 | 9 | 1 |
|  | P-value | - | .02 | .39 | .08 | .01 | .85 |
|  | Mean Lifespan [days] | 20.8 | 19.3 | 19.9 | 22.0 | 22.6 | 21.0 |
|  | Number of animals | 63 | 81 | 69 | 59 | 55 | 74 |
| N2 | Change in lifespan [%] | - | -5 | -3 | 6 | 30 | 14 |
|  | P-value | - | .29 | .21 | 4.8E-3 | 6.0E-15 | 1.1E-5 |
|  | Mean Lifespan [days] | 27 | 25.8 | 26.2 | 28.6 | 35.0 | 30.8 |
|  | Number of animals | 71 | 69 | 72 | 71 | 73 | 67 |

All experiments conducted with γ-irradiated dead bacteria (OP50)
