# Supplementary material for: Translation attenuation by minocycline enhances longevity and proteostasis in old post-stress-responsive organisms
Source: eLife. 2018 Nov 27;7:e40314. doi: 10.7554/eLife.40314 (PMC6257811; doi:10.7554/eLife.40314)
Supplement: Supplementary file 1. [file elife-40314-supp1.docx]

| **PMID** | **PMID** | **process** | **Model** |
| --- | --- | --- | --- |
| Minocycline alleviates beta-amyloid protein and tau pathology via restraining neuroinflammation induced by diabetic metabolic disorder | 23983461 | aggregation | Diabetic rats |
| Doxycycline attenuates protein aggregation in cardiomyocytes and improves survival of a mouse model of cardiac proteinopathy | 20947000 | aggregation | CryAb TG mice |
| Minocycline corrects early, pre-plaque neuroinflammation and inhibits BACE-1 in a transgenic model of Alzheimer's disease-like amyloid pathology | 22472085 | aggregation | McGill-Thy1-APP mice |
| Minocycline reduces the development of abnormal tau species in models of Alzheimer’s disease | 19001528 | aggregation | Htau mice |
| Minocycline neuroprotects, reduces microgliosis, and inhibits caspase protease expression early after spinal cord injury | 16638021 | aggregation | Sprague-Dawley rat*s* |
| Minocycline attenuates neuronal cell death and improves  cognitive impairment in Alzheimer’s disease models | 17406652 | aggregation | Tg2576 mice |
| Minocycline prevents cholinergic loss in a mouse model of Down’s syndrome | 5468085 | aggregation | Ts65Dn mice |
| Minocycline affects microglia activation, Abeta deposition, and behavior in APP-tg mice | 16534778 | aggregation | APP-tg mice |
| Inhibition of thrombin-induced microglial activation and NADPH oxidase by minocycline protects dopaminergic neurons in the substantia nigra in vivo | 16219027 | aggregation / inflamm. | Rat thrombin injection |
| Increases in β-amyloid protein in the hippocampus caused by diabetic metabolic disorder are blocked by minocycline through inhibition of NF-kB pathway | 21602593 | aggregation / inflamm. | Diabetic rats |
| Early-stage inflammation and experimental therapy in transgenic models of the Alzheimer-like amyloid pathology | 0173335 | aggregation / inflamm | AD-like tg mice |
| Minocycline reduces inflammatory parameters in the brain structures and serum and reverses memory impairment caused by the administration of amyloid β (1-42) in mice | 28336494 | inflammation | BALB/c mice |
| The intestinal anti-inflammatory effect of minocycline in experimental colitis involves both its immunomodulatory properties | 21193045 | inflammation | TNBS-induced rat colitis/ DSS-induced mouse colitis |
| Minocycline attenuates lipopolysaccharide (LPS)-induced neuroinflammation, sickness behavior, and anhedonia | 18477398 | inflammation | BALB/c mice |
| Minocycline attenuates experimental colitis in mice by blocking expression of inducible nitric oxide synthase and matrix metalloproteinase | 19285099 | inflammation | TNBS/DSS-induced mouse colitis |
| Minocycline reduces proinflammatory cytokine expression, microglial activation, and caspase-3 activation in a rodent model of diabetic retinopathy. | 15855346 | inflammation | SD-rats |
| Tetracyclines inhibit microglial activation and are neuroprotective in global brain ischemia | 9861045 | inflammation | Gerbils |
| Multiple neuroprotective mechanisms of minocycline in autoimmune CNS inflammation | 17239606 | inflammation | MOG-induced autoimmune encephalomyelitis rat |
| A tetracycline derivative, minocycline, reduces inflammation and protects against focal cerebral ischemia with a wide therapeutic window | 10557349 | inflammation | Sprague–Dawley rats |

**Supplementary File 1. Literature on minocycline and other tetracycline derivatives studied for their beneficial effects on inhibiting protein aggregation and amyloid formation.**
