# Supplementary material for: Translation attenuation by minocycline enhances longevity and proteostasis in old post-stress-responsive organisms
Source: eLife. 2018 Nov 27;7:e40314. doi: 10.7554/eLife.40314 (PMC6257811; doi:10.7554/eLife.40314)
Supplement: Supplementary file 2. [file elife-40314-supp2.docx]

**Supplementary File 2. Completed and ongoing clinical trials with minocycline for indications relating to its neuroprotective, anti-inflammatory and additional beneficial effects.**

| **Trial** | **NCT** |
| --- | --- |
| A Phase II Study of Minocycline and Armodafinil for Reducing the Symptom Burden Produced by Chemoradiation Treatment for Esophageal Cancer | 01746043 |
| Combined Treatment of Minocycline and Lovastatin to Treat Individuals With Fragile X Syndrome | 02680379 |
| Evaluation of Oral Minocycline in the Treatment of Geographic Atrophy Associated With Age-Related Macular Degeneration | 02564978 |
| Minocycline Plus Amiodarone Versus Amiodarone Alone for the Prevention of Atrial Fibrillation After Cardiac Surgery | 01422148 |
| Minocycline in Primary Sclerosing Cholangitis (PSC) | 00630942 |
| Study of Oral Minocycline in Treating Bilateral Cystoid Macular Edema Associated With Retinitis Pigmentosa | 02140164 |
| Study of a Neuroprotective Drug to Limit the Extent of Damage From an Ischemic Stroke | 00630396 |

**NCT**: A unique identification code given to each clinical study record registered on ClinicalTrials.gov.
