# Supplementary material for: Translation attenuation by minocycline enhances longevity and proteostasis in old post-stress-responsive organisms
Source: eLife. 2018 Nov 27;7:e40314. doi: 10.7554/eLife.40314 (PMC6257811; doi:10.7554/eLife.40314)
Supplement: Transparent reporting form [file elife-40314-transrepform.pdf]

1st Floor
24 Hills Road
Cambridge CB2 1JP, UK

P 01223 855340
W elifesciences.org
T @elife

eLife's transparent reporting form

We encourage authors to provide detailed information *within their submission* to facilitate the interpretation and replication of experiments. Authors can upload supporting documentation to indicate the use of appropriate reporting guidelines for health-related research (see EQUATOR Network), life science research (see the BioSharing Information Resource), or the ARRIVE guidelines for reporting work involving animal research. Where applicable, authors should refer to any relevant reporting standards documents in this form.

If you have any questions, please consult our Journal Policies and/or contact us: editorial@elifesciences.org.

Sample-size estimation

- You should state whether an appropriate sample size was computed when the study was being designed
- You should state the statistical method of sample size computation and any required assumptions
- If no explicit power analysis was used, you should describe how you decided what sample (replicate) size (number) to use

Please outline where this information can be found within the submission (e.g., sections or figure legends), or explain why this information doesn't apply to your submission:

The sample sizes to ensure sufficient power of detection for all lifespan experiments were determined using the methods in Petrascheck and Miller (PMID 28713422). To do this we generated a non-parametric model based on our own lifespan data and ran monte-carlo simulations using various assumptions and evaluated them using Mantel-Haenzel version of the log rank test.

Replicates

- You should report how often each experiment was performed
- You should include a definition of biological versus technical replication
- The data obtained should be provided and sufficient information should be provided to indicate the number of independent biological and/or technical replicates
- If you encountered any outliers, you should describe how these were handled
- Criteria for exclusion/inclusion of data should be clearly stated
- High-throughput sequence data should be uploaded before submission, with a private link for reviewers provided (these are available from both GEO and ArrayExpress)

Please outline where this information can be found within the submission (e.g., sections or figure legends), or explain why this information doesn't apply to your submission:

The number of times each experiment was replicated is indicated in the figure legend as “total of X independent experiments” with the exact numbers of n in the supplementary tables. Only biological replicates conducted. Our definition of an independent experiment / biological replicate is an experiment conducted by a different researcher or an experiments started at a different date requiring the fresh preparation of all biological entities (cells, worms, bacteria) to run the experiment
Outlier handling: Each cohort in our lifespan experiments are conducted using 8-16 small sub cohorts of 3-12 animals each. Our computational analysis pipeline routinely tests for outlier cohorts and allows for only one sub-cohort to be removed if the lifespan of the sub-cohort is significantly ($P<0.01$) different from all other sub-cohorts that were treated identically. Each removal is automatically logged in non-editable file and the analysis cannot proceed unless the researcher doing the analysis makes a decision whether or not to include the outlier. In our statistical pipeline outliers are defined as outside of the 99% CI and are expected at a rate of 1%. If more than the expected rate (1%) of outliers is observed (determined by a binomial probability test), the experiment was evaluated for general problems such as contamination, DMSO oxidation, insufficient bacterial food and similar. If such an event was deemed to have occurred, the entire experiment was declared invalid and repeated. If the expected rate of outliers is detected, the default is not to remove any data and to proceed with the analysis encompassing the entire dataset. This strategy of outlier testing not only detects potential problems with the over-all experiment, but also is an excellent quality control mechanism to determine the general assumption used in statistics that everything else but the intervention has been the same.
Our publication contains a very limited amount of –omics data regarding the ABPP, for which there is no real database and small-scale proteomics (~400 proteins) to evaluate translation based on isotope incorporation. Both files are uploaded with the manuscript.

Statistical reporting

- Statistical analysis methods should be described and justified
- Raw data should be presented in figures whenever informative to do so (typically when N per group is less than 10)
- For each experiment, you should identify the statistical tests used, exact values of N, definitions of center, methods of multiple test correction, and dispersion and precision measures (e.g., mean, median, SD, SEM, confidence intervals; and, for the major substantive results, a measure of effect size (e.g., Pearson's r, Cohen's d)
- Report exact p-values wherever possible alongside the summary statistics and 95% confidence intervals. These should be reported for all key questions and not only when the p-value is less than 0.05.

Please outline where this information can be found within the submission (e.g., sections or figure legends), or explain why this information doesn't apply to your submission:

Figure 1: GFP scatter plots A: Analysis was conducted using one way ANOVA followed by either the Dunett's test if we compared to a single control, or the t-test if a sample was only compared to a single other control sample (e.g. treated vs untreated). Due to the large numbers of animals used (n=100-300) testing using different approaches had not effect on the outcome.
Lifespan C, E: Mantel Hanezel version of the log-rank test the standard non-parametric test for longevity data. Using the Mantel-Cox proportional hazard method generally reports identical results with regards to significance. However, the assumption of a proportional hazard is not appropriate for many longevity studies.
Protein aggregation Fig.1 H: For each time point a one way ANOVA was conducted followed by a Dunett's test comparing against untreated control.

Figure 2B, C: GFP scatter plots, see Figure 1, A,B.
Lifespan D, E: same as Figure 1, C, E

Fig. 3:
A: Statistical analysis for the enrichment of certain GO annotation is a built in function in the cytoscape plug in ClueGO and reports a hypergeometric P value.
B, C: t-test comparing water vs minocycline treatment
D: Statistical significance between the proteomic runs was determined using a paired t-test comparing the level of each protein (dot) in the treated or untreated samples.
Figure 4: D: qPCR data were evaluated using ANOVA followed by dunett's test. f: t-test comparing treated and untreated.
Fig. 5: see lifespan data

(For large datasets, or papers with a very large number of statistical tests, you may upload a single table file with tests, Ns, etc., with reference to sections in the manuscript.)

Group allocation

- Indicate how samples were allocated into experimental groups (in the case of clinical studies, please specify allocation to treatment method); if randomization was used, please also state if restricted randomization was applied
- Indicate if masking was used during group allocation, data collection and/or data analysis

Please outline where this information can be found within the submission (e.g., sections or figure legends), or explain why this information doesn't apply to your submission:

Allocation of worms was random either using pipetting or a COPAS BIOSORT.
Experiments were not conducted blind, but nearly all experiments were repeated by a second researcher.

Additional data files ("source data")

- We encourage you to upload relevant additional data files, such as numerical data that are represented as a graph in a figure, or as a summary table
- Where provided, these should be in the most useful format, and they can be uploaded as "Source data" files linked to a main figure or table
- Include model definition files including the full list of parameters used
- Include code used for data analysis (e.g., R, MatLab)
- Avoid stating that data files are "available upon request"

Please indicate the figures or tables for which source data files have been provided:

For lifespan data we have uploaded tables detailing each experiment individually
Raw data for the –omics studies were uploaded as excels
The full-length gels were included in the supplement for all experiments
Statistical analysis were conducted either using STATA or graph pad.
